# Editorial: Social inequality and equity in community actions for health

**DOI:** 10.3389/fpubh.2023.1144910

**Published:** 2023-02-13

**Authors:** Mobolanle Balogun, Aduragbemi Banke-Thomas, Shannon Galvin, Godfred O. Boateng

**Affiliations:** ^1^Department of Community Health and Primary Care, College of Medicine of the University of Lagos, Lagos, Nigeria; ^2^School of Human Sciences, University of Greenwich, London, United Kingdom; ^3^Department of Infectious Diseases and Epidemiology, London School of Hygiene and Tropical Medicine, London, United Kingdom; ^4^Department of Medicine, Northwestern University Feinberg School of Medicine, Chicago, IL, United States; ^5^School of Global Health, York University, Toronto, ON, Canada

**Keywords:** social inequalities, equity, access, community action, community participation

Health inequities are systematic differences in the health status of different population groups (1). These differences are widening between and within countries with such concern that the World Health Organization used World Health Day 2021 to mark health equity and commenced a year-long equity campaign to bring people together to build a fairer, healthier world (2). The causes of health inequity stem from a range of social, economic, environmental, and structural disparities that result in differences in health outcomes across populations (3). The Coronavirus Disease 2019 (COVID-19) pandemic has further illuminated and magnified health inequities in both high-, middle- and low-income countries (4, 5), reversing progress made over the last 20 years. Consequently, we launched this Research Topic on the 21^st^ of September 2021 with the aim of cataloging articles that document health inequality and inequity globally as well as articles that address both through community action.

Of the manuscripts that were submitted, we eventually accepted and published 10 which fall into four research areas: (1) relationship between social inequality and health outcomes, (2) community actions among socially vulnerable groups, (3) role of health professionals in addressing health inequity in communities and (4) new concepts in defining health disparities.

There is a gradient between socioeconomic status and health with each level in the hierarchy generally having less morbidity and mortality. For some health conditions, however, there has been no change in health or worsening health status over time for economically disadvantaged populations (6). Holder-Pearson and Chase in their opinion article describe how certain marginalized ethnic and socioeconomic groups in New Zealand bear a disproportionately high burden of Type 2 diabetes mellitus, suffer higher financial costs of care and have lower access to life-saving treatment. Contrarily, two studies in our topic did not elicit negative health outcomes among populations with social disadvantages. First, Chan et al. in their review article show that individuals experiencing homelessness and traumatic brain injuries in studies from United States of America and Canada had rehabilitation services available to them. They recommend that existing rehabilitation for these individuals should be tailored to include screening for TBI, conducting cognitive and functional assessments and involve multidisciplinary teams. Second, Hamilton et al. in their single-center retrospective study of 73 children with medical complexities presenting with sepsis, did not find any association between social determinants of health and length of stay in the pediatric intensive care unit.

Community actions play a vital role in promoting health equity, as they occur at a level closer to individuals and can be better targeted at high-risk individuals. Each community is unique in the nature and degree of health inequities as well the required community-based efforts (7). Mishra et al. used a participatory learning action technique to formatively assess community participation in a rural, vulnerable population in India and developed a conceptual framework for community participation while Hoffman et al. engaged community experts and organizations working in refugee, immigrant and migrant communities and explored their perspectives and roles in the COVID-19 pandemic response.

Regarding the role of health professionals in addressing health inequity in communities, Hurley-Kim et al. outline the health disparities that exist in pharmacists' practice in the United States, including communities with limited access to pharmacies (pharmacy deserts) and innovative solutions proposed by pharmacy leaders to address the disparities. Chong et al. share how community pharmacists in Malaysia manage medication wastage, returned medicines, and medicines disposal while Li et al. describe the role and challenges of village doctors in rural China during the prevention and control of the COVID-19 pandemic. These articles underscore the central role health care professionals have in addressing health inequity. Indeed, a previous study includes provider distribution according to population need and practice patterns oriented to addressing root causes of disparities as some of the critical domains to advancing health equity (8).

Two new concepts feature in the fourth area of research. The first by Dierx and Kasper details the development of a new grouping to measure socio-economic status, providing new insights into health inequalities. This is critical since advancement of health equity requires a proper assessment of differences in health and its determinants (9). Development of structured formats of measurements for different societies is deemed necessary (10). The second by Ju et al. proposes a new model for the process of rumor diffusion about COVID-19 and they recommend announcing true information publicly to instantly contain the COVID-19 rumor diffusion.

In conclusion, our Research Topic brought together multiple scientific disciplines to catalog social inequality, health inequity, community and health care professionals' actions and innovation to advance health equity. The COVID-19 pandemic has highlighted the relevance of community-based efforts to advance health equity. Most of our studies were cross-sectional; further studies that use randomized control trials and/or longitudinal data are recommended to establish causal relationships.

## Author contributions

All authors listed have made a substantial, direct, and intellectual contribution to the work and approved it for publication.
